# Progress towards the UNAIDS 2030 HIV prevention target in New South Wales, Australia: a population-based study

**DOI:** 10.1016/j.lanwpc.2025.101535

**Published:** 2025-04-12

**Authors:** Phillip Keen, Steven J. Nigro, Curtis Chan, Benjamin R. Bavinton, Htein Linn Aung, Martin Holt, Rebecca Guy, Janaki Amin, Timothy R. Broady, Jane Costello, Anthony D. Kelleher, Carla Treloar, Rick Varma, Matthew Vaughan, Valerie Delpech, Andrew E. Grulich

**Affiliations:** a The Kirby Institute, University of New South Wales, Sydney, NSW, Australia; b New South Wales Ministry of Health, Sydney, NSW, Australia; c Centre for Social Research in Health, University of New South Wales, Sydney, NSW, Australia; d Department of Health Science, Faculty of Medicine Health and Human Sciences, Macquarie University, Sydney, NSW, Australia; e Positive Life NSW, Sydney, NSW, Australia; f Sydney Sexual Health Centre, Sydney, NSW, Australia; g ACON, Sydney, NSW, Australia

**Keywords:** HIV prevention, HIV testing, Pre-exposure prophylaxis, HIV treatment, Undetectable viral load, Gay and bisexual men

## Abstract

**Background:**

The UNAIDS ending AIDS strategy includes a 2030 prevention target of a 90% reduction in new infections from 2010. We report progress towards this goal in gay, bisexual and other men who have sex with men (GBM) in New South Wales (NSW), Australia.

**Methods:**

We report HIV notification data for people newly diagnosed by exposure category, with a focus on GBM who comprised more than three-quarters of diagnoses. We report HIV testing, pre-exposure prophylaxis, HIV treatment, and undetectable viral load based on surveys of community-based GBM and data from a sentinel surveillance network of 50 clinics. We report trends between 2010 and 2022, including by geography grouped by postcodes with high-, medium- and low-prevalence of gay residents. Trends were assessed using the chi-square test for linear trend.

**Findings:**

Statewide, annual notifications declined by 56% in GBM, and declines were much greater in inner-Sydney postcodes with a high percentage of gay residents compared to postcodes with a low percentage (88% and 32%). Among community-recruited GBM, annual HIV testing and PrEP uptake increased over time and by 2022 were higher in the high- (91% and 82%) than low-gay prevalence postcodes (78% and 61%). In the clinic sample, HIV testing and PrEP use increased but there was no evidence that they differed by geography. In both samples, among GBM living with HIV, the percentages on HIV treatment and with undetectable viral load increased over time, and by 2022 were greater than 95%.

**Interpretation:**

HIV notifications in GBM in NSW have dropped by more than half since 2010. In inner Sydney areas with a high prevalence of gay men, prevention uptake was highest, and the decline in notifications approached 90%. Declines in HIV notifications were more modest elsewhere, and prevention uptake lower. Currently available prevention interventions, if extended population-wide, can enable a 90% reduction in new HIV infections in GBM, consistent with the ending AIDS target.

**Funding:**

This project was funded by the 10.13039/501100000925National Health and Medical Research Council and the 10.13039/501100008810NSW Ministry of Health.

**Research in context**

**Evidence before this study**

In March 2024 we conducted a search of the literature to identify articles on population-based trends in HIV diagnoses in countries, and in large cities, to compare trends against the UNAIDS target of a 90% reduction of HIV diagnoses between 2010 and 2030. We used combinations of relevant keywords (ie HIV, AIDS, epidemic, prevention, elimination, ending, population) in Pub Med. As many reports of HIV surveillance data are not published in the peer-reviewed literature, we also used Google searches to identify grey literature. We sought to identify English language articles published between January 1 2020 and January 1 2024 which reported population-based trends in HIV diagnoses.

We identified several reports describing declines in modelled estimates of HIV incidence. In eight African countries (Zimbabwe, Lesotho, Rwanda, Eritrea, Eswatini, Malawi, Burundi and Ethiopia), and in Nepal, the decline between 2022 and 2010 was 70% or greater, indicating they are on-track to reach the UNAIDS target. We identified a number of high-income settings with high HIV testing rates which reported trends in HIV notifications. At the country level, the largest declines were seen in Europe (more than 50% decline between 2010 and 2021 in Denmark, England and the Netherlands). Larger declines were seen in a small number of large cities including Amsterdam, San Francisco, and London.

**Added value of this study**

We assessed HIV notification trends in Australia's most populous state, New South Wales (NSW), with a focus on gay and bisexual men (GBM) and progress towards UNAIDS targets. Based on emerging surveillance data, we examined trends separately for the parts of Sydney where the concentration of gay men was highest. We compared geographical trends with corresponding trends in uptake of HIV prevention interventions including HIV testing, HIV treatment and pre-exposure prophylaxis.

Statewide, between 2010 and 2022 there was a 51% decline in HIV notifications (56% in GBM). The decline in GBM was greatest in the parts of inner Sydney with the highest concentration of GBM (>20%), with an 88% decline, approaching the UNAIDS target of 90%. Areas with the lowest concentration of GBM (<5%) had only a 32% decline. Similarly, the uptake of HIV prevention was greatest in the parts of inner-Sydney with >20% prevalence of GBM. In this area, among HIV negative GBM who reported condomless sex, 91% reported HIV testing in the last year and 82% reported PrEP use. Among GBM living with HIV, more than 95% were receiving HIV treatment. Levels of PrEP use and HIV testing were much lower in the low-prevalence areas.

**Implications of all the available evidence**

Data from NSW represent real-world evidence that the UNAIDS target of a 90% reduction in HIV diagnoses since 2010 can be reached if currently available interventions are used at very high levels. Population wide achievement of UNAIDS goals will require considerable scale up over wider geographic areas, requiring tailoring of the response to local population needs.

## Introduction

Two major advances in HIV prevention research have led to the proposition that the AIDS epidemic can be ended as a “global public health threat”.^1^ First, it was demonstrated that people on HIV treatment with undetectable viral load cannot sexually transmit HIV.^2^, ^3^, ^4^ Second, HIV pre-exposure prophylaxis (PrEP) in adherent individuals has been shown to be almost 100% effective in preventing HIV acquisition.^5^^,^^6^ The World Health Organization (WHO) defines “elimination as a public health threat” as the achievement of measurable targets requiring continued action.^7^ In 2014, UNAIDS released its ending AIDS strategy which included a prevention target of a 90% reduction in new annual HIV infections between 2010 and 2030.^1^ Although progress against this target was initially slow, with UNAIDS declaring a “global prevention crisis” in 2018,^8^ subsequent strategies have maintained the 2030 prevention target.^9^ In 2020, the government of Australia's most populous state, New South Wales (NSW), released a 2021–2025 strategy proposing a 90% reduction in the rate of HIV notifications, compared to a 2008 to 2012 baseline, in line with the UNAIDS target.^10^

In this report, we examine statewide and geographically-localised trends in NSW HIV notifications between 2008 and 12 and 2022, with a focus on gay, bisexual and other men who have sex with men (GBM) who comprise more than three-quarters of diagnoses.^11^^,^^12^ To contextualise trends in HIV notifications, we separately examine trends before and after COVID-19, and present complementary data on prevention indicators, including HIV testing, PrEP use, HIV treatment, and undetectable viral load.

## Methods

### Data sources and measures

Data on HIV notifications between 2008 and 2022 were sourced from the NSW Notifiable Conditions Information Management System. The NSW *Public Health Act 2010* requires mandatory reporting of HIV diagnoses from laboratories and medical practitioners. Diagnoses previously made outside of NSW were excluded. We examined characteristics of people notified with HIV including gender, HIV exposure history, previous HIV testing history, CD4 count and clinical status at diagnosis, history of symptoms consistent with HIV seroconversion illness at diagnosis, and residential postcode. Cases were classified as recent HIV infections where there was a clinical diagnosis of seroconversion illness or a negative or indeterminate HIV test within 12 months before diagnosis.^11^ Trends in recent HIV infections may be a more accurate reflection of trends in HIV incidence than all diagnoses.^13^ We use the term “gay, bisexual and other men who have sex with men” (GBM) to be maximally inclusive of men who may be exposed to HIV through sex with another man. We acknowledge that individuals may choose to adopt different terms to describe their sexual orientation and sexual behaviour.

We report HIV prevention indicator data on HIV testing, PrEP use, HIV treatment and undetectable viral load in GBM from separate community- and clinic-based systems as outlined in Table 1. First, HIV prevention data in community-recruited GBM were sourced from the Sydney Gay Community Periodic Survey (SGCPS). The SGCPS is an annually repeated cross-sectional HIV behavioural surveillance survey conducted among GBM aged 16 and over with recruitment at venues, bars, clubs, events, and clinics (since 1996) and online (since 2014). The 2020 survey was completed before COVID-19 affected Australia and in 2021 recruitment was entirely conducted online.^14^ Second, HIV prevention data from GBM attending clinics were sourced from NSW sites in the Australian Collaboration for Coordinated Enhanced Sentinel Surveillance (ACCESS) Network. ACCESS extracts de-identified routinely-collected electronic medical record data from clinic patient management systems. As individuals may attend multiple clinics, data are linked across the network of health services using probabilistic and deidentified linkage keys.^15^ For this study, we included data on GBM aged 16 years or older attending 34 publicly-funded sexual health clinics, ten private general practice clinics with medium to high caseloads of GBM, two hospital outpatient clinics, and four peer-led community-based testing services.^15^ GBM status was identified through sexuality variables, or through a proxy (male gender and a history of rectal sexually transmitted infection (STI) tests).^16^

**Table 1:** HIV prevention indicators measured in the community-recruited and clinic-based samples among gay and bisexual men who had indicators that they would have been high-risk for HIV transmission if effective HIV prevention was not used^a^.

Indicator	Community-recruited sample (data based on self-report in the Sydney Gay Community Periodic Survey)	Clinic-based sample (data electronically extracted from clinical records from at least one NSW clinic in the ACCESS Network)
HIV testing	Percentage of participants who were not living with HIV who reported having one or more HIV tests in the 12 months prior to the survey	Percentage of HIV-negative patients who had at least one HIV test in the previous 12 months
HIV pre-exposure prophylaxis (PrEP)	Percentage of participants who were not living with HIV who reported use of PrEP in the 6 months prior to the survey	Percentage of HIV-negative patients who were recorded as being on PrEP at least once during the calendar year
HIV treatment	Percentage of participants living with HIV who reported being on combination antiretroviral treatment for HIV	Percentage of people living with HIV who were recorded as being on HIV treatment in the previous 12 months
Undetectable viral load	Percentage of participants living with HIV who reported that their last HIV viral load test result was undetectable	Proportion of people living with HIV who were recorded as having undetectable HIV viral load (<200 RNA copies/mm^3^) at their most recent test in the previous 12 months

PrEP = pre-exposure prophylaxis.

a In the community-recruited sample, the indicator of high-risk for HIV transmission was defined as reporting condomless anal intercourse with casual male partners. In the clinic-based sample, the indicator of high-risk for HIV transmission was having more than 20 sexual partners in the past 12 months and not using condoms consistently. Where behavioural data were not available, we defined this as having a diagnosis of a rectal STI in the past 2 years, or injecting drug use in the last 12 months.

We report HIV prevention indicator trends for GBM classified as “high-risk”. For the community-recruited sample, we defined this as those who reported condomless anal intercourse with casual male partners. For the clinic-based sample, based on available data, we defined this as having more than 20 sexual partners in the past 12 months and not using condoms consistently. Where behavioural data were not available, we defined this as having a diagnosis of a rectal STI in the past 2 years, or injecting drug use in the last 12 months.^17^

### Analysis

#### Geographic analyses

**Fig. 1:**
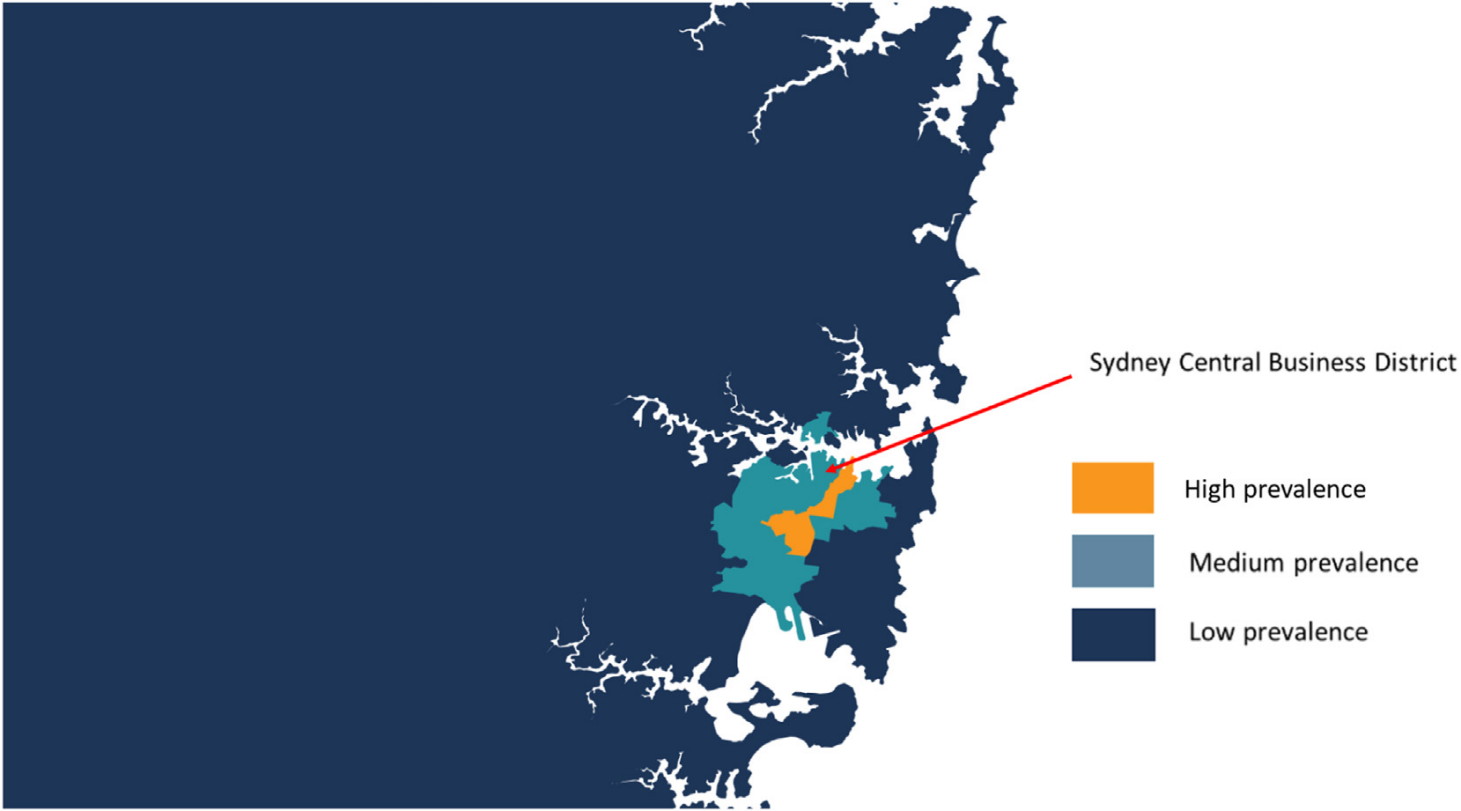
**Map of Sydney indicating the location of high-, medium-, and low-gay prevalence areas**. High prevalence = postcodes where ≥20% of adult males are estimated to be gay; Medium prevalence = postcodes where 5–19·9% of adult males are estimated to be gay; Low prevalence = postcodes where <5% of adult males are estimated to be gay. In all other postcodes in the remainder of the Sydney metropolitan area and New South Wales (not shown on map) the estimated gay population prevalence is <5%.^12^

Our geographic analyses were based on published estimates of the percentage of adult males in each postcode who were estimated to be gay. These estimates were derived from the 2016 Australian Census (the approximate mid-point of our analyses), and surveys of Australian gay men conducted between 2011 and 2017.^12^ We then grouped these estimates into three area categories: (i) high prevalence (≥20% gay residents, six postcodes in inner Sydney), (ii) medium prevalence (5–19·9% gay residents, twenty-four postcodes around inner Sydney), and (iii) low prevalence (<5% gay residents, elsewhere in Sydney and NSW; Fig. 1).^10^^,^^17^ We stratified HIV notifications and HIV prevention indicator trends by these three areas. For the most recent year of data collection (2022) we examined whether HIV prevention uptake varied across the three geographical areas using chi-square tests for linear trend and the goodness of fit test for the linear trend model.

#### Time trends

For HIV notifications, we compared annual HIV notifications in 2013–2022 to the mean number of notifications in the five-year period 2008–2012, the baseline specified in the NSW HIV Strategy.^10^ This five-year baseline comparator was used to account for annual fluctuations in the number of diagnoses in NSW, and because the mid-point (2010) was the baseline used for UNAIDS targets.^9^ For all notifications, we report both numbers of notifications and rates per 100,000, using the total NSW population as the denominator. We calculated percentage decline in notification rates, and confidence intervals based on the Poisson distribution. The study had 90% power to detect a significant decrease in HIV notification rate of 23% or greater. The absence of population denominators meant that we could not report incidence rates for people with different risk exposures. For HIV prevention indicators, in the community-recruited sample we used data collected between 2008 and 2022 except for PrEP use (only available from 2015). In the clinic-based sample, the first year of data availability was 2009. The significance of time trends was assessed using the chi-square test for linear trend, as above, for the period from when data were available until the last pre-COVID-19 year, which was 2020 for the community-recruited sample (data collected in February before COVID-19 affected Australia) and 2019 for the clinic-based sample. To assess potential associations with COVID-19, we compared the last pre-COVID-19 year with the final year of data availability (2022) with statistical significance calculated using the chi-square test for difference, or the Fisher's exact test when the expected count in any cell was less than 5. A p-value of <0·05 was deemed statistically significant. All analyses were conducted using Stata (version 14.2; College Station, TX).^18^

### Ethic approvals

The SGCPS has ethics approval from the UNSW Sydney Human Research Ethics Committee (HC180903, 13/12/2018). Information collected was entirely anonymous and therefore completing the questionnaire was taken as consent. The ACCESS Network has ethics approval from the Human Research Ethics Committee of Alfred Hospital (248/17, 4/8/2017). Informed consent was provided by participating services, with individual patient consent waived due to the de-identified nature of the collected data.

### Role of the funding source

As a formal partnership research project, the NSW Ministry of Health had an active role in the design, conduct and analyses reported here, and several NSW Ministry of Health staff were included as manuscript co-authors.

## Results

### Trends in HIV diagnoses

Between 2008 and 2022, 4503 people were newly diagnosed with HIV in NSW, of whom 3518 (78%) reported male to male sexual contact (hereafter termed gay, bisexual and other men who have sex with men, GBM), 787 (17%) reported heterosexual contact, and 100 (2%) reported injecting drug use. Compared to the five-year mean of diagnoses between 2008 and 2012 (343), annual new diagnoses declined overall by 51% to 167 by 2022, and the population rate per 100,000 fell by 58% (95% CI 49–65%, p < 0·0001; Table 2). Diagnoses among GBM and heterosexual people declined by 56% (268 to 119), and 34% (58 to 38) respectively. Overall, 1821 (40%) of diagnoses were classified as recent HIV infections. Recent HIV infections declined by 67% overall (from 151 to 50, with a decline in the population rate of 71%, 95% CI 60%–79%), and by 69% and 50% in GBM and heterosexuals, respectively (Table 2).

**Table 2:** Number of new HIV diagnoses in New South Wales by HIV risk exposure: five-year mean of the period 2008–2012, and annual diagnoses between 2013 and 2022 a) for all diagnoses and b) for diagnoses with evidence of recent HIV infection.

HIV risk exposure	Baseline (mean, 2008–2012)	2013	2014	2015	2016	2017	2018	2019	2020	2021	2022	Decline between baseline and 2022, n, (%)
**All HIV diagnoses**												
Male to male sexual exposure	267·6	282	274	285	262	232	219	216	155	136	119	157·6 (55·5%)
Heterosexual sexual exposure	57·8	60	50	52	48	68	51	56	40	35	38	19·8 (34·3%)
People who inject drugs	10·2	7	8	4	4	6	4	5	3	4	4	6·2 (60·8%)
Other or unknown	7·8	5	11	8	4	7	3	4	8	3	6	1·8 (23·1%)
**Total (n)**	**343·4**	**354**	**343**	**349**	**318**	**313**	**277**	**281**	**206**	**178**	**167**	**176·8 (51·4%)**
**Population rate per 100,000**	**4**·**8**	**4**·**8**	**4**·**6**	**4**·**6**	**4**·**1**	**4**·**0**	**3·5**	**3**·**5**	**2**·**5**	**2**·**2**	**2**·**0**	**57**·**5%**
**HIV diagnoses with evidence of recent HIV infection**												
Male to male sexual exposure	137·6	136	143	141	132	93	94	83	59	42	43	94·6 (68·8%)
Heterosexual sexual exposure	12	10	11	9	11	12	12	9	4	4	6	6 (50·0%)
People who inject drugs	1·4	1	2	0	1	1	0	0	0	0	1	0·4 (28·6%)
Other or unknown	0·8	0	3	1	0	0	1	0	0	0	0	0·8 (100%)
**Total (n)**	**151·2**	**147**	**159**	**151**	**144**	**106**	**107**	**92**	**63**	**46**	**50**	**101·2 (66·9%)**
**Population rate per 100,000**	**2·1**	**2·0**	**2·1**	**2·0**	**1·9**	**1·3**	**1·3**	**1·1**	**0·78**	**0·57**	**0·61**	**(71%)**

Population rates per 100,000 are provided for total population.

Data are numbers of cases, population rates per 100,000, or number of cases and percentage change.

**Fig. 2:**
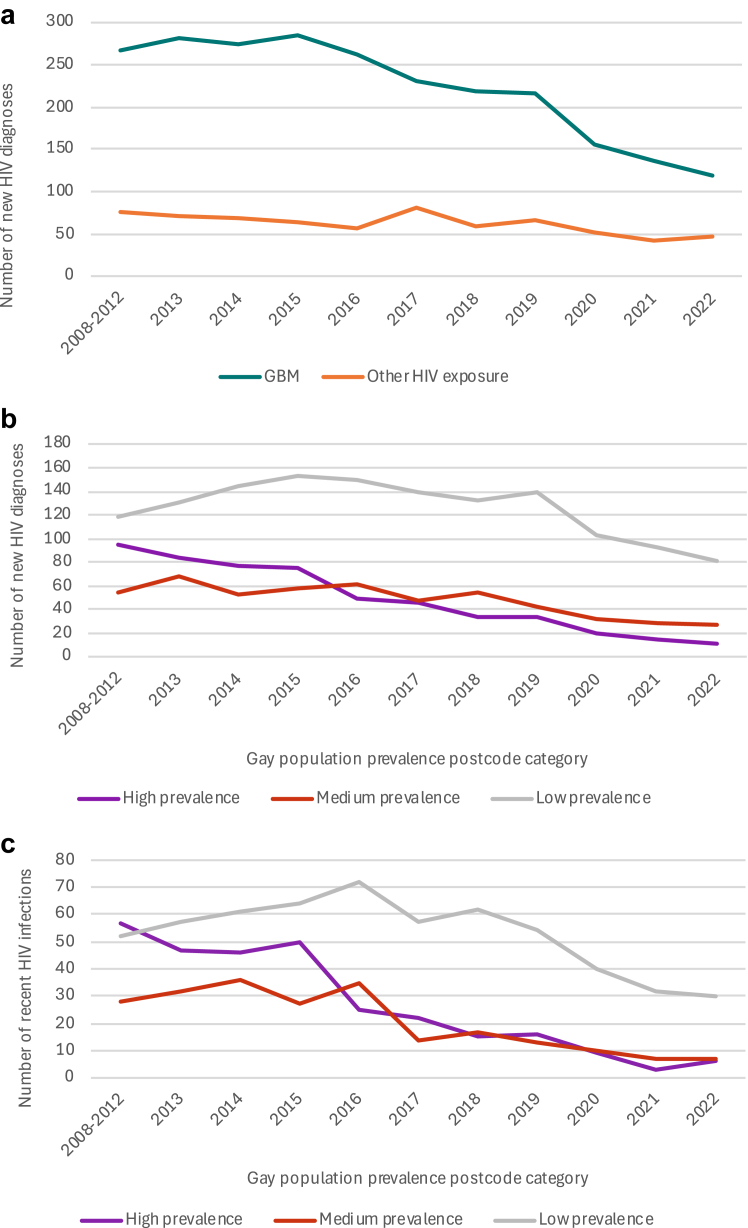
**Number of new HIV diagnoses, New South Wales 2008–2012 (five-year mean) and annually 2013 to 2022 (a)** All new HIV diagnoses among gay, bisexual and other men who have sex with men (GBM), and all new diagnoses among people with other HIV exposures. **(b)** New HIV diagnoses among GBM in NSW by gay population prevalence postcode category. **(c)** New HIV diagnoses among GBM with evidence of recent HIV infection in NSW, by gay population prevalence postcode category.

Notifications in GBM declined more in high-gay prevalence areas. By 2022, diagnoses among GBM declined by 88% (95 to 11), 50% (54 to 27) and 32% (119 to 81) respectively in high-, medium-, and low-gay prevalence areas (Fig. 2b, Table 3). Even greater declines were observed with recent infection: 89% (57 to 6), 75% (28 to 7), and 43% (52 to 30) in the three areas (Fig. 2c, Table 3).

**Table 3:** HIV diagnoses and HIV diagnoses with evidence of recent HIV infection among gay, bisexual and other men who have sex with men in New South Wales, by area of residence according to gay population prevalence postcode category: five-year mean of the period 2008–2012, and annual diagnoses between 2013 and 2021.

Gay population prevalence postcode category	Mean annual HIV diagnoses 2008–2012 (baseline)	2013	2014	2015	2016	2017	2018	2019	2020	2021	2022	Decline between baseline and 2022 n, (%)
**All HIV diagnoses in gay and bisexual men**												
Low prevalence (<5%)	119·0	131	145	153	150	139	132	139	103	93	81	38 (31·9%)
Medium prevalence (5–19%)	53·8	68	53	57	62	48	54	43	32	29	27	26·8 (49·8%)
High prevalence (≥20%)	94·8	83	76	75	50	45	33	34	20	14	11	83·8 (88·4%)
**HIV diagnoses with evidence of recent HIV infection among GBM**												
Low prevalence (<5%)	52·4	57	61	64	72	57	62	54	40	32	30	22·4 (42·7%)
Medium prevalence (5–19%)	28·4	32	36	27	35	14	17	13	10	7	7	21·4 (75·4%)
High prevalence (≥20%)	56·8	47	46	50	25	22	15	16	9	3	6	50·8 (89·4%)

Data are numbers of cases, or number of cases and percentage change.

### HIV testing in the previous 12 months among non HIV-positive GBM

**Fig. 3:**
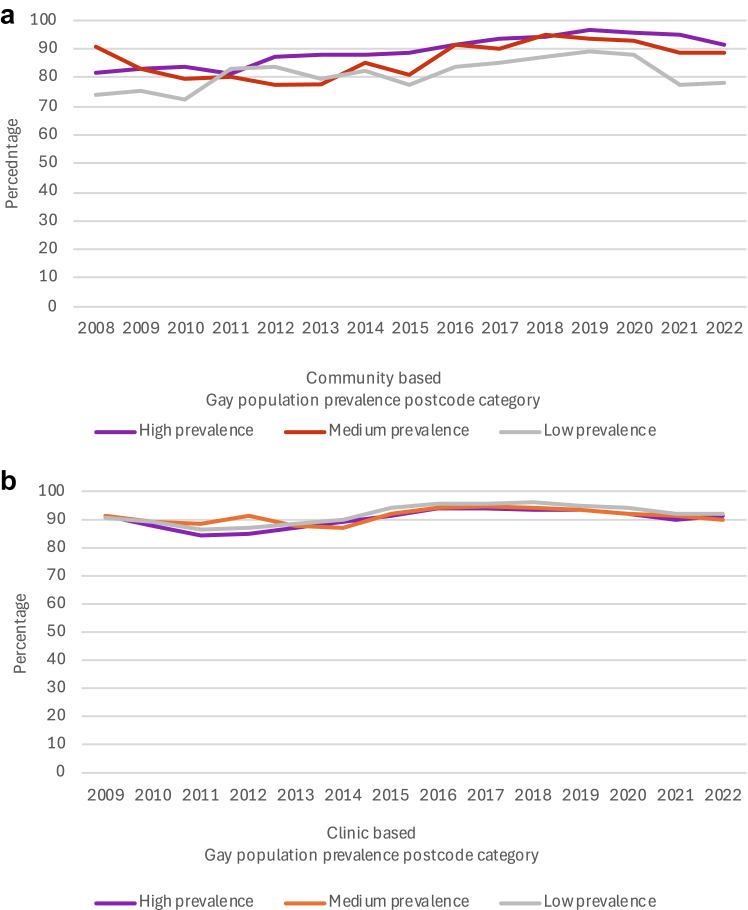
**HIV testing among non HIV-positive gay and bisexual men who reported high-risk behaviours in New South Wales 2008/9–2022 in community-based and clinic-based settings, by gay population prevalence (a)** Percentage who reported one or more HIV test in the previous twelve months in the Sydney Gay Community Periodic Surveys. **(b)** Percentage receiving HIV testing at least once in the previous 12 months at any NSW clinic in the ACCESS Network, 2009 to 2022.

In the community-recruited sample, among those who reported high risk behaviours, the percentage who reported at least one HIV test in the previous 12 months rose significantly from 82% (224/275) in 2008 to 92% (995/1080) in 2020 (p-trend<0·0001), and testing increased in each gay prevalence area (p-trend<0·0001 in each; Fig. 3a, Table S1-A). Then, between 2020 and 2022, the percentage tested fell from 92% to 84% (606/720, p < 0·0001) overall and fell in the high and low gay prevalence areas (p = 0·027, and p < 0·0001). In 2022, HIV testing was substantially more common in men from higher-gay prevalence areas (p*-*trend<0·0001).

In the clinic-based sample, among those categorized as high risk, the percentage who received at least one HIV test in the previous twelve months rose from 91% (754/829) in 2009 to 94% (7352/7807) in 2019 (p-trend<0·0001) and testing increased in each gay prevalence area (p-trend < 0·0001 in each; Fig. 3b, Table S1-B). Then, between 2019 and 2022, the percentage tested fell from 94% to 91% overall (5295/5796, p < 0·0001), including in each gay prevalence area (p = 0·039 in high, and p < 0·0001 in medium and low). In 2022, there was no evidence that HIV testing differed across the three areas (p-trend = 0·30).

### PrEP use among non HIV-positive GBM

**Fig. 4:**
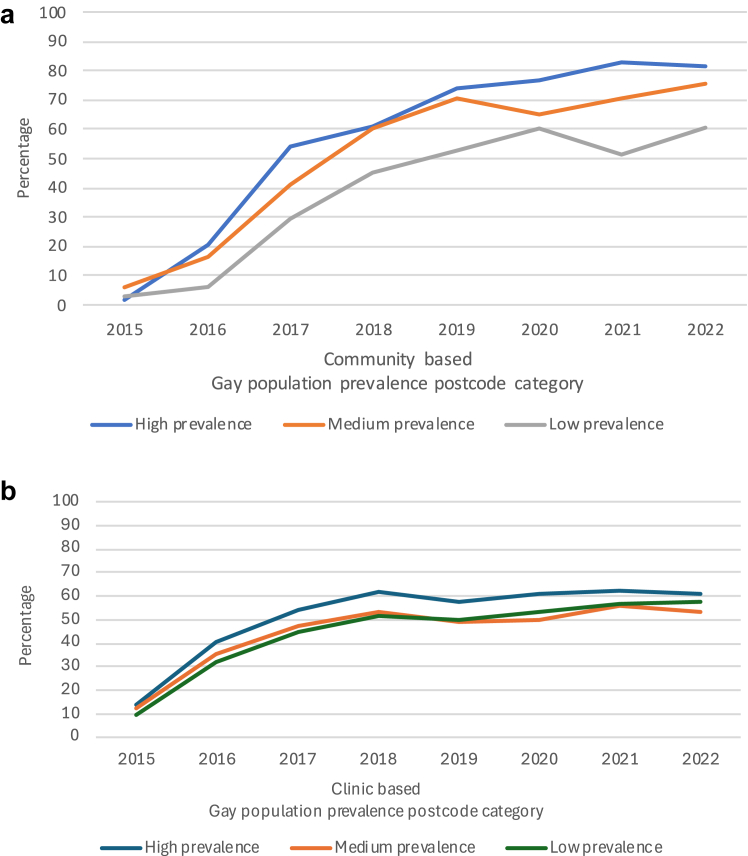
**HIV pre-exposure prophylaxis (PrEP) use among non HIV-positive gay and bisexual men who reported high-risk behaviours in New South Wales 2015–2022 in community-based and clinic-based settings by gay population prevalence (a)** Percentage who reported use of HIV pre-exposure prophylaxis (PrEP) in the last six months in the Sydney Gay Community Periodic Surveys. **(b)** Percentage who were recorded as being on PrEP at least once in the calendar year at any NSW clinic in the ACCESS Network, 2015 to 2022.

In the community-recruited sample, among those who reported high risk behaviours, the percentage who reported using PrEP in the previous six months rose from 3% (13/406) in 2015 to 67% (721/1069) in 2020 (p-trend < 0·0001) and increased in each gay prevalence area (p-trend < 0·0001 in each, Fig. 4a, Table S2-A). Then, between 2020 and 2022, PrEP use overall was stable. PrEP use was stable in the high- and low prevalence areas (p = 0·15 and p = 0·88) and increased in the medium prevalence area (65% (160/245) to 76% (139/184), p = 0·022). In 2022, PrEP use was substantially more common in the high and medium prevalence areas (p-trend < 0·0001).

In the clinic-based sample, among those categorized as high risk, the percentage recorded as being on PrEP at least once during the calendar year rose from 12% (522/4434) in 2015 to 52% (4039/7807) in 2019 (p-trend < 0·0001) and PrEP use increased in each gay prevalence area (p-trend < 0·0001 in each, Fig. 4b, Table S2-B). Then, between 2019 and 2022, PrEP use increased from 52% to 57% (3312/5796) overall (p < 0·0001) and increased significantly in each gay prevalence area (p = 0·040, 0·024 and p < 0·0001 in high, medium and low). In 2022, there was no evidence that PrEP use differed across gay prevalence areas (p-trend = 0·16).

### HIV treatment among GBM living with HIV

**Fig. 5:**
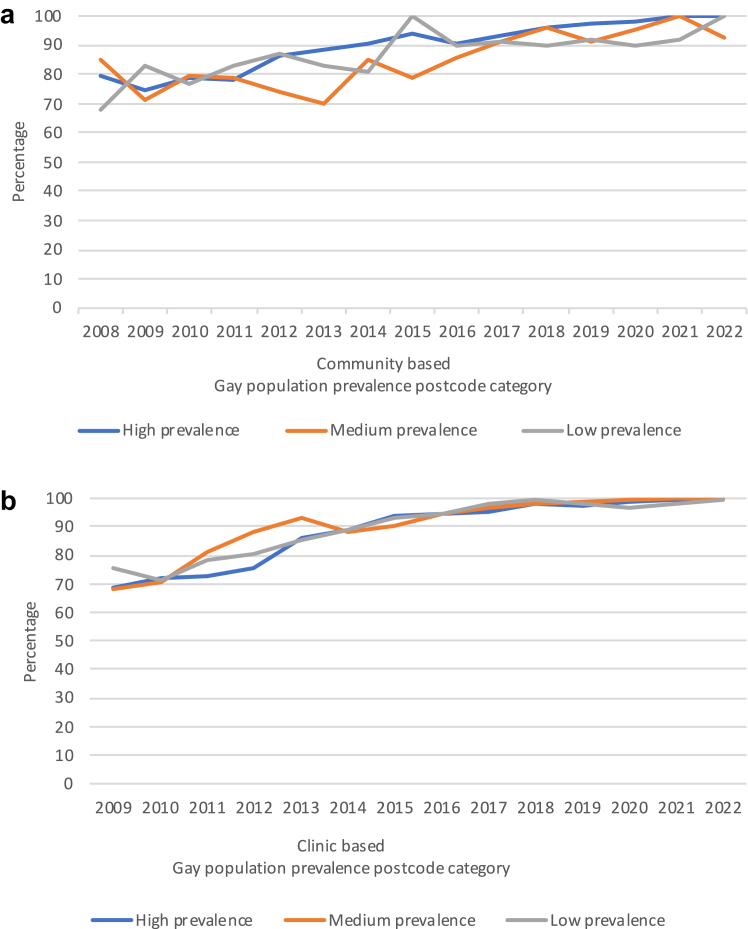
**HIV treatment use among gay and bisexual men living with HIV who reported high-risk behaviours in New South Wales 2008/9–2022 in community-based and clinic-based settings by gay population prevalence (a)** Percentage who reported being on treatment in the Sydney Gay Community Periodic Surveys; **(b)** Percentage attending ACCESS clinics who were recorded as being on treatment in the past 12 months in the ACCESS Network.

In the community-recruited sample, among those who reported high risk behaviours, the overall percentage who reported being on HIV treatment rose from 78% (89/114) in 2008 to 95% (107/113) in 2020 (p-trend < 0·0001) and increased in each gay prevalence area (p-trend < 0·0001 in each, Fig. 5a, Table S3-A). Then, between 2020 and 2022, there was no change in the percentage on treatment overall (p = 0·42) and in each gay prevalence area (p = 1·00, p = 1·00, and p = 0·13 in the high-, medium-, and low-areas, respectively). In 2022, there was no evidence that percentage on treatment varied by gay prevalence area (p-trend = 0·16).

In the clinic-based sample, among those categorized as high risk, HIV treatment coverage rose from 71% (193/272) in 2009 to 98% (807/822) in 2019 (p-trend < 0·001) and increased in each gay prevalence area (p-trend<0·001 in each, Fig. 5b, Table S3-B). Then, between 2019 and 2022, the overall percentage on HIV treatment continued to rise (98%–100% (524/525), p = 0·007), but there was no evidence of significant change in each gay prevalence area (p = 0·053, 0·14, and 0·20 in the high-, medium- and low-areas, respectively). In 2022 there was no evidence that percentage on treatment varied by area (100% in each, p-trend = 0·29).

### Undetectable viral load among GBM living with HIV

**Fig. 6:**
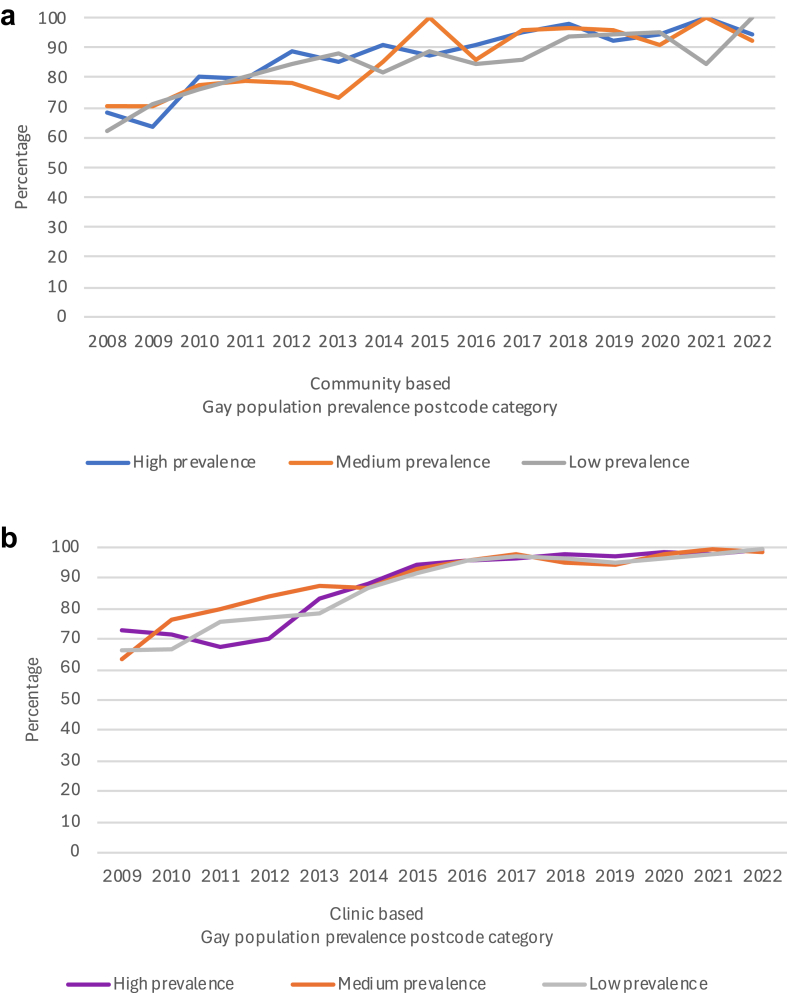
**Undetectable viral load among gay and bisexual men living with HIV who reported high-risk behaviours in New South Wales 2008/9–2022 in community-based and clinic-based settings by gay population prevalence (a)** Percentage who reported that their last HIV viral load test result was undetectable in the Sydney Gay Community Periodic Surveys. **(b)** Percentage attending ACCESS NSW clinics who were recorded as having undetectable HIV viral load at their most recent test in the previous 12 months in the ACCESS Network.

In the community-recruited sample, among those who reported high risk behaviours, the percentage who reported that their last viral load test was undetectable rose from 67% (75/112) in 2008 to 94% (107/114) in 2020 (p-trend < 0·0001) and the percentage increased in each gay prevalence area (p-trend < 0·0001 in each, Fig. 6a, Table S4-A). Then, between 2020 and 2022, the percentage with an undetectable viral load remained stable overall (p = 0·72) and in each gay prevalence area (p = 1·00, p = 1·00, and p = 0·51 in the high-, medium- and low-areas, respectively, Fig. 6a, Table S4-A), In 2022 there was no evidence that the percentage with an undetectable viral load varied by area (p-trend = 0·38, Fig. 6a, Table S4-A).

In the clinic-based sample, among those categorized as high risk, the percentage with an undetectable viral load rose from 68% (184/269) in 2009 to 96% (759/795) in 2019 (p-trend < 0·0001) and in each gay prevalence area (p-trend < 0·0001 in each, Fig. 6b, Table S4-B). Then, between 2019 and 2022, the percentage with an undetectable viral load continued to increase from 96% to 99% (518/522) overall (p < 0·0001) and in each gay prevalence area (p = 0·13, 0·032, and 0·003, in high-, medium- and low-areas respectively). In 2022, there was no evidence that percentage with undetectable viral load varied by area (99% in high and medium, and 100% in low, p-trend = 0·78).

## Discussion

HIV notifications among GBM in inner Sydney areas with a high prevalence of gay men declined by 88% between 2008 and 12 and 2022. Thus, by this indirect measure of HIV transmission, inner Sydney has almost reached the UNAIDS target of a 90% reduction in HIV infections between 2010 and 2030.^9^ Outside of inner Sydney, in areas with low-gay prevalence, HIV diagnoses declined by only 32% (38 cases). Statewide, the decline in HIV diagnoses among GBM was 56% (158 cases), with a substantially larger decline (69%, 95 cases) in recent HIV infection. This geographic pattern was reflected in similar variations in the uptake of preventive interventions. In our community-recruited sample, in HIV-negative men who reported recent condomless anal intercourse, between 2008 and 2022 HIV testing in the last 12 months increased from 82% (89/109) to 91% (169/185) in the high-gay prevalence area and from 74% (68/92) to 78% (272/349) in the low-gay prevalence area. Since 2015, PrEP use by GBM who reported condomless sex increased from 2% (2/117) to 82% (148/181), and 3% (6/206) to 61% (210/347), in the high- and low-gay prevalence areas, respectively. In the most recent year (2022) there was lower uptake of both HIV testing and PrEP in the low-gay prevalence areas. Among GBM living with HIV, treatment uptake increased markedly over this time period (from 78% (89/114) to 98% (59/60)) and this varied little geographically. In our clinic-based system, there were similar levels of uptake of HIV testing and PrEP across geographic areas, suggesting that once connected to clinical care, uptake levels were similar regardless of geography.

Substantial declines in HIV diagnoses in GBM have been described in several other high-income settings in recent years. In Europe, declines in new HIV diagnoses in GBM of at least 50% have been described in Denmark, England, the Netherlands and Scotland.^19^, ^20^, ^21^, ^22^ The largest nationwide decline reported was 68% in the Netherlands between 2010 and 2021.^21^ In England, the decline between 2017 and 2021 was 53%.^20^ In North America, there was a 35% decline in Canada (2011–2020)^23^ and in the USA there was a 12% decline in estimated infections (2017–2021).^24^ Declines of a larger magnitude than the national estimates have been described in certain large cities including London (68%, between 2017 and 2021),^25^ San Francisco (77%, between 2012 and 2021)^26^ and Amsterdam (83%, between 2010 and 2022),^27^ demonstrating that larger declines in HIV have been achieved in smaller geographic areas with concentrated GBM populations. In Africa, based on modelled data, UNAIDS estimates that there are eight PEPFAR-supported countries with generalized HIV epidemics which have experienced a 70% or greater decline in estimated annual new HIV infections between 2010 and 2022.^28^ Trends in key populations including GBM are not presented in these modelled estimates.

During the COVID-19 pandemic there was a faster decline in HIV notifications in NSW. Between 2019 and 2022, there was a 36% decline in HIV diagnoses in GBM, mostly due to a 50% decline in HIV diagnoses in overseas-born GBM.^29^ The closure of Australia's borders between March 2020 and February 2022 played an important role, with very few migrants entering the country. From March 2022, migration to Australia rebounded quickly, and in the 12 month period from July 1 2022, net overseas migration reached an all-time high.^30^ Accompanying the rebound in migration, during 2023 there was a 70% increase in HIV diagnoses in overseas-born GBM in NSW, compared to a 5% increase in Australia-born GBM, and almost two-thirds (65%) of total HIV diagnoses in GBM were in the overseas-born.^29^ There were other COVID-19 associated factors which may have led to fewer HIV diagnoses between 2019 and 2022. Stay-at-home orders and physical distancing restrictions implemented from March 2020 were associated with declining sexual risk behaviour and PrEP use.^14^ In our community-based data, we documented substantial drops in HIV testing which were greater in the low-than the high-gay prevalence areas, and in 2022 testing levels rebounded but remained below pre-COVID-19 levels. PrEP use initially declined dramatically in the low-gay prevalence areas, but by 2022 uptake had returned to pre-COVID-19 levels. In 2022, PrEP uptake remained highest in the high-prevalence areas, intermediate in medium prevalence, and lowest in the low-prevalence area. Among those who attended clinics, COVID-19-associated changes in HIV testing and PrEP use were much smaller, but the total number of clinic visits declined substantially. Levels of HIV treatment and undetectable viral load did not change appreciably during COVID-19 in any location, likely related to innovations such as the widespread uptake of telehealth.

There are a number of factors which have contributed to NSW's success in HIV prevention among GBM. Australia has a universal healthcare system which provides citizens and permanent residents affordable access to healthcare and medicines. In addition, NSW has a statewide network of 52 sexual health clinics which provide free sexual healthcare to GBM and other priority populations, regardless of Australian residency status. The HIV prevention response in NSW is guided by HIV strategies which outline clear, measurable targets in HIV testing, PrEP, prevention coverage,^31^ and for HIV diagnosis and treatment.^10^ Reporting against these targets occurred quarterly.^11^ For GBM, there have been intensive efforts to increase the uptake and frequency of HIV testing, including by making testing easier at sexual health clinics and through community-based services.^32^ The current strategy targets 90% PrEP use by GBM who report condomless anal intercourse with casual partners.^10^ Many of the initiatives targeting GBM, including health promotion, demand-creation and community-based testing, have been led by a well-established and trusted statewide HIV and LGBTQ health organization, ACON, and a statewide community-based organization for people living with HIV, Positive Life NSW. The success of inner Sydney, with a close to 90% reduction in annual HIV diagnoses, is likely partly explained by the higher concentration of gay-friendly clinical and prevention HIV services in the inner Sydney areas where GBM live. This is likely amplified by the fact that social engagement with other gay men is strongly associated with uptake of HIV prevention interventions in Australia.^33^

In our setting of high HIV testing rates and a long-standing mandatory HIV notification system, we believe that the HIV diagnosis trends we have described likely reflect trends in incidence. A recently reported cohort study of almost 60,000 GBM at 69 clinics in NSW and Victoria reported a decline in directly-measured HIV incidence of 66% between 2010 and 2019,^34^ somewhat greater than the 56% decline in HIV diagnoses but very close to the 69% decline in recent HIV infection that we documented in NSW surveillance data. As population dominators were unavailable, we could not calculate the per-capita incidence rate in GBM. As the estimated male population increased by 16% between 2010 and 2022, it is likely that incidence rates declined by even more than the decline in notifications.^35^ The declines in HIV testing in 2020–22 that we documented may have contributed towards the faster decline in HIV diagnoses during the COVID-19 period. However, persistently low HIV diagnoses in Australian-born GBM in 2023 despite HIV testing rates which have rebounded towards pre-COVID-19 levels^29^ suggests that less HIV testing cannot explain the declining HIV diagnosis rates in the Australian-born. Our community-recruited and clinic-based data systems on uptake of HIV testing, PrEP and treatment were based on different samples and had differing strengths and weaknesses. The community-based system provides a community-wide measure of the use of HIV testing and prevention but is based on self-report and is likely to have over-sampled more community-involved GBM. The clinic system only includes service-engaged GBM, and data were extracted from clinical records The ACCESS Network covers the majority of sexual health services and high GBM caseload general practices in NSW; however, it does not capture all GBM living in NSW. Based on the data available, the definition of high-risk was different, and this limits the comparability of the two systems. Sexuality information was not collected at general practices and GBM status is approximated with a proxy of a rectal STI test, which may under or overestimate GBM at general practices.

The 88% decline in HIV diagnoses among GBM who live in postcodes of inner Sydney with the highest levels of prevention uptake is a real-world demonstration that reaching the UNAIDS 2030 target of a 90% reduction in HIV infections is feasible with currently available interventions. Dramatic reductions in HIV diagnoses in GBM in a few European and North American cities suggest a similar pattern.^25^, ^26^, ^27^ Our data also reveal a remaining challenge of smaller reductions in HIV diagnoses, and lower levels of prevention uptake, outside of inner Sydney. Achieving higher prevention uptake statewide will require tailored strategies to overcome barriers, particularly in culturally and linguistically diverse communities. In 2023, the increase in HIV diagnoses in overseas-born GBM, which accompanied record-high levels of immigration, is a clear demonstration of how Australia's HIV epidemic is connected internationally. Extending inner-Sydney's successes in HIV prevention in GBM countrywide and across all populations at risk is the next major challenge for the HIV response in Australia.

## Contributors

AG led this study in partnership with VD, PK and SN. All authors contributed to its design, development, and implementation. Funding was secured by AG, BB, TB, RG, MH, AK, SN, CT and MV. The methods were designed by AG, BB, VD, RG, PK and SN. Data Analysis was performed by PK and AG and data interpretation by AG, HA, BB, CC, VD, RG, MH, PK and SN. The manuscript was drafted by AG, PK, SN and VD. PK and SN contributed equally as first authors, and AG and VD contributed equally as senior authors. All authors participated in review and editing. All authors gave final approval of the version to be published and agree to be accountable for all aspects of the work in ensuring that questions related to the accuracy or integrity of any part of the work are appropriately investigated and resolved. AG and PK accessed and verified the underlying data reported in the manuscript.

## Data sharing statement

Researchers may request access to data collected by the ACCESS project by submitting an analysis plan for review and agreeing to adhere to security, confidentiality, and collaborative conditions. More information can be found by visiting https://accessproject.org.au/data. Researchers may request access to limited, deidentified data from the Gay Community Periodic Surveys chief investigators by submitting a concept sheet and analysis plan for review and agreeing to adhere to security, confidentiality, and collaborative conditions. HIV surveillance data are routinely collected and protected according to NSW and Australian legislation, and cannot be shared. Requests for release of de-identified unit record data can be made to Health Protection NSW and require review and approval by the NSW Chief Health Officer.

## Declaration of interests

PK has received research funding to his institution from the NSW Ministry of Health, personal fees and travel support from Gilead Sciences. BB has received research funding to his institution from Viiv Health Care, Gilead Sciences, NHMRC and ACON, personal fees from FHI360 and Gilead Sciences, and travel support from Virology Education and Viiv Health Care. MH has received research funding to his institution from the NSW Ministry of Health. RG has received research funding to her institution from Cepheid and SpeeDx. TB has received a personal fee from Gilead Sciences. AK has received research funding to his institution from the NHMRC, the Australian Department of Foreign Affairs and Trade, and the US National Institutes of Health. MV reported receiving funding to his organisation from the NSW Ministry of Health and grants to his organisation from Viiv Health Care and Gilead Sciences. AG has received research funding to his institution from the NSW Ministry of Health, GSK, and ViiV Health Care, travel funding from ViiV Health Care and in-kind research support from GSK. SN, CC, HLA, JA, JC, CT, VD report no COI.
