# Supplementary material for: Progress towards the UNAIDS 2030 HIV prevention target in New South Wales, Australia: a population-based study
Source: Lancet Reg Health West Pac. 2025 Apr 12;57:101535. doi: 10.1016/j.lanwpc.2025.101535 (PMC12019848; doi:10.1016/j.lanwpc.2025.101535)
Supplement: Supplementary Tables [file mmc1.docx]

**SUPPLEMENTARY APPENDIX**

Contents

[**Section S1:** Community and clinic-collected data on HIV testing among high-risk GBM in New South Wales 2](#_Toc171334073)

[**Table S1-A:** HIV testing in the previous 12 months among non-HIV positive high-risk GBM (i.e., those who reported condomless anal intercourse with casual partners in the previous six months) overall, and by residence in gay population prevalence postcode category, Sydney Gay Community Periodic Surveys 2008-2022 2](#_Toc171334074)

[**Table S1-B:** HIV testing in the previous 12 months at any clinic in the ACCESS NSW Network among high-risk GBM patients overall, and by residence in gay population prevalence postcode category, ACCESS NSW Network 2009-2022 3](#_Toc171334075)

[**Section S2:** Community and clinic-collected data on PrEP use among high-risk GBM in New South Wales 4](#_Toc171334076)

[**Table S2-A:** PrEP use in the previous 6 months among non-HIV positive high-risk GBM (i.e., those who reported condomless anal intercourse with casual partners in the previous six months) overall, and by residence in gay population prevalence postcode category, Sydney Gay Community Periodic Surveys 2008-2022 4](#_Toc171334077)

[**Table S2-B:** Proportion of high-risk HIV-negative GBM patients recorded as being on PrEP, overall, and by gay population prevalence postcode category, ACCESS NSW Network 2015-2022 5](#_Toc171334078)

[**Section S3:** Community and clinic-collected data on HIV treatment among high-risk HIV positive GBM in New South Wales 6](#_Toc171334079)

[**Table S3-A:** HIV treatment among HIV positive high-risk GBM (i.e., those who reported condomless anal intercourse with casual partners in the previous six months) overall, and by residence in gay population prevalence postcode category, Sydney Gay Community Periodic Surveys 2008-2022 6](#_Toc171334080)

[**Table S3-B*:*** Proportion of high-risk HIV-positive GBM patients recorded on HIV treatment, overall, and by gay population prevalence postcode category, ACCESS NSW Network 2009-2022 7](#_Toc171334081)

[**Section S4:** Community and clinic-collected data on undetectable viral load among high-risk HIV positive GBM in New South Wales 8](#_Toc171334082)

[**Table S4-A*:*** Proportion of high-risk HIV-positive GBM (i.e., those who reported condomless anal intercourse with casual partners in the previous six months) who reported that the result of their last HIV viral load test was undetectable, overall, and by residence in gay population prevalence postcode category, Sydney Gay Community Periodic Surveys 2008-2022 8](#_Toc171334083)

[**Table S4-B:** Proportion of high-risk HIV-positive GBM patients recorded as having undetectable viral load, overall, and by gay population prevalence postcode category, ACCESS NSW Network 2009-2022 9](#_Toc171334084)

# ***Section S1:*** *Community and clinic-collected data on HIV testing among high-risk GBM in New South Wales*

## **Table S1-A:** HIV testing in the previous 12 months among non-HIV positive high-risk GBM (i.e., those who reported condomless anal intercourse with casual partners in the previous six months) overall, and by residence in gay population prevalence postcode category, Sydney Gay Community Periodic Surveys 2008-2022

|  | **Year** | | | | | | | | | | | | | | | |  |
| --- | --- | --- | --- | --- | --- | --- | --- | --- | --- | --- | --- | --- | --- | --- | --- | --- | --- |
|  | **2008** | **2009** | **2010** | **2011** | **2012** | **2013** | **2014** | **2015** | **2016** | **2017** | **2018** | **2019** | **2020** | **p-trend*** | **2021** | **2022** | **p-value†** |
| **Overall (n)** | 275 | 358 | 827 | 448 | 382 | 394 | 353 | 473 | 594 | 861 | 848 | 1001 | 1080 | ·· | 623 | 720 | ·· |
| One or more tests (n) | 224 | 288 | 651 | 366 | 320 | 325 | 301 | 385 | 523 | 767 | 778 | 932 | 995 | ·· | 519 | 606 | ·· |
| One or more tests (%) | 81·5 | 80·4 | 78·7 | 81·7 | 83·8 | 82·5 | 85·3 | 81·4 | 88·0 | 89·1 | 91·7 | 93·1 | 92·1 | <0·0001 | 83·3 | 84·2 | <0·0001 |
| **Low prevalence (n)** | 92 | 130 | 304 | 160 | 124 | 137 | 123 | 234 | 263 | 364 | 348 | 390 | 424 | ·· | 359 | 349 | ·· |
| One or more tests (n) | 68 | 98 | 220 | 133 | 104 | 109 | 101 | 181 | 220 | 309 | 305 | 348 | 373 | ·· | 277 | 272 | ·· |
| One or more tests (%) | 73·9 | 75·4 | 72·4 | 83·1 | 83·9 | 79·6 | 82·1 | 77·4 | 83·7 | 84·9 | 87·6 | 89·2 | 88·0 | <0·0001 | 77·2 | 77·9 | <0·0001 |
| **Medium prevalence (n)** | 74 | 114 | 208 | 105 | 99 | 103 | 96 | 101 | 148 | 197 | 201 | 246 | 248 | ·· | 130 | 186 | ·· |
| One or more tests (n) | 67 | 95 | 166 | 84 | 77 | 80 | 82 | 82 | 135 | 178 | 191 | 231 | 231 | ·· | 115 | 165 | ·· |
| One or more tests (%) | 90·5 | 83·3 | 79·8 | 80·0 | 77·8 | 77·7 | 85·4 | 81·2 | 91·2 | 90·4 | 95·0 | 93·9 | 93·1 | <0·0001 | 88·5 | 88·7 | 0·11 |
| **High prevalence (n)** | 109 | 114 | 315 | 183 | 159 | 154 | 134 | 138 | 183 | 300 | 299 | 365 | 408 | ·· | 134 | 185 | ·· |
| One or more tests (n) | 89 | 95 | 265 | 149 | 139 | 136 | 118 | 122 | 168 | 280 | 282 | 353 | 391 | ·· | 127 | 169 | ·· |
| One or more tests (%) | 81·7 | 83·3 | 84·1 | 81·4 | 87·4 | 88·3 | 88·1 | 88·4 | 91·8 | 93·3 | 94·3 | 96·7 | 95·8 | <0·0001 | 94·8 | 91·4 | 0·027 |
| **p-trend‡** | ·· | ·· | ·· | ·· | ·· | ·· | ·· | ·· | ·· | ·· | ·· | ·· | ·· | ·· |  | <0.0001 | ·· |

Low prevalence, medium prevalence, and high prevalence=residence in low, medium and high gay population prevalence postcodes categories, respectively. *p-trend for time trend 2008-2020; **†**p-value test for difference between 2020 and 2022; ‡p-trend for difference between gay prevalence postcode categories in 2022.

## **Table S1-B:** HIV testing in the previous 12 months at any clinic in the ACCESS NSW Network among high-risk GBM patients overall, and by residence in gay population prevalence postcode category, ACCESS NSW Network 2009-2022

|  | **Year** | | | | | | | | | | | | | | |  |
| --- | --- | --- | --- | --- | --- | --- | --- | --- | --- | --- | --- | --- | --- | --- | --- | --- |
|  | **2009** | **2010** | **2011** | **2012** | **2013** | **2014** | **2015** | **2016** | **2017** | **2018** | **2019** | **p-trend*** | **2020** | **2021** | **2022** | **p-value†** |
| **Overall (n)** | 829 | 1138 | 1314 | 1692 | 2334 | 3285 | 4434 | 5497 | 6717 | 7561 | 7807 |  | 6679 | 5645 | 5796 |  |
| One or more tests (n) | 754 | 1009 | 1132 | 1478 | 2052 | 2922 | 4114 | 5211 | 6382 | 7174 | 7352 |  | 6227 | 5150 | 5295 |  |
| One or more tests (%) | 90·9 | 88·6 | 86·1 | 87·3 | 87·9 | 88·9 | 92·7 | 94·8 | 95% | 94·8 | 94·1 | <0·0001 | 93·2 | 91·2 | 91·3 | <0·0001 |
| **Low prevalence (n)** | 279 | 376 | 451 | 574 | 879 | 1301 | 1752 | 2238 | 2870 | 3297 | 3595 |  | 3215 | 2540 | 2662 |  |
| One or more tests (n) | 252 | 336 | 390 | 498 | 779 | 1173 | 1650 | 2144 | 2755 | 3171 | 3417 |  | 3032 | 2331 | 2454 |  |
| One or more tests (%) | 90·3 | 89·3 | 86·4 | 86·7 | 88·6 | 90·1 | 94·1 | 95·8 | 95·9 | 96·1 | 95·0 | <0·0001 | 94·3 | 91·7 | 92·1 | <0·0001 |
| **Medium prevalence (n)** | 207 | 314 | 344 | 464 | 626 | 885 | 1179 | 1416 | 1756 | 1995 | 2048 |  | 1637 | 1514 | 1599 |  |
| One or more tests (n) | 189 | 279 | 304 | 423 | 549 | 770 | 1086 | 1335 | 1662 | 1880 | 1914 |  | 1512 | 1383 | 1435 |  |
| One or more tests (%) | 91·3 | 88·8 | 88·3 | 91·1 | 87·7 | 87·0 | 92·1 | 94·2 | 94·6 | 94·2 | 93·4 | <0·0001 | 92·3 | 91·3 | 89·7 | <0·0001 |
| **High prevalence (n)** | 343 | 448 | 519 | 654 | 829 | 1099 | 1503 | 1843 | 2091 | 2269 | 2164 |  | 1827 | 1591 | 1535 |  |
| One or more tests (n) | 313 | 394 | 438 | 557 | 724 | 979 | 1378 | 1732 | 1965 | 2123 | 2021 |  | 1683 | 1436 | 1406 |  |
| One or more tests (%) | 91·2 | 87·9 | 84·3 | 85·1 | 87·3 | 89·0 | 91·6 | 93·9 | 93·9 | 93·5 | 93·3 | <0·0001 | 92·1 | 90·2 | 91·6 | 0·039 |
| **p-trend‡** |  |  |  |  |  |  |  |  |  |  |  |  |  |  | 0·30 |  |

Low prevalence, medium prevalence, and high prevalence=residence in low, medium and high gay population prevalence postcodes categories, respectively. *p-trend for time trend 2009-2019; **†**p-value test for difference between 2019 and 2022; ‡p-trend for difference between gay prevalence postcode categories in 2022.

# ***Section S2:*** *Community and clinic-collected data on PrEP use among high-risk GBM in New South Wales*

## **Table S2-A:** PrEP use in the previous 6 months among non-HIV positive high-risk GBM (i.e., those who reported condomless anal intercourse with casual partners in the previous six months) overall, and by residence in gay population prevalence postcode category, Sydney Gay Community Periodic Surveys 2008-2022

|  | **Year** | | | | | | | | |  |
| --- | --- | --- | --- | --- | --- | --- | --- | --- | --- | --- |
|  | **2015** | **2016** | **2017** | **2018** | **2019** | **2020** | **p-trend*** | **2021** | **2022** | **p-value†** |
| **Overall (n)** | 406 | 512 | 774 | 733 | 924 | 1069 | ·· | 623 | 632 | ·· |
| On PrEP (n) | 13 | 67 | 315 | 398 | 599 | 721 | ·· | 386 | 417 | 0·54 |
| On PrEP (%) | 3·2 | 13·1 | 40·7 | 54·3 | 64·8 | 67·4 | <0·0001 | 62·0 | 66·0 | ·· |
| **Low prevalence (n)** | 206 | 229 | 330 | 303 | 357 | 420 | ·· | 359 | 347 | ·· |
| On PrEP (n) | 6 | 14 | 98 | 137 | 187 | 252 | ·· | 183 | 210 | ·· |
| On PrEP (%) | 2·9 | 6·1 | 29·7 | 45·2 | 52·4 | 60·0 | <0·0001 | 51·0 | 60·5 | 0·88 |
| **Medium prevalence (n)** | 83 | 121 | 179 | 173 | 226 | 245 | ·· | 130 | 184 | ·· |
| On PrEP (n) | 5 | 20 | 74 | 104 | 160 | 160 | ·· | 92 | 139 | ·· |
| On PrEP (%) | 6·0 | 16·5 | 41·3 | 60·1 | 70·8 | 65·3 | <0·0001 | 70·8 | 75·5 | 0·022 |
| **High prevalence (n)** | 117 | 162 | 265 | 257 | 341 | 404 | ·· | 134 | 181 | ·· |
| On PrEP (n) | 2 | 33 | 143 | 157 | 252 | 309 | ·· | 111 | 148 | ·· |
| On PrEP (%) | 1·7 | 20·4 | 54·0 | 61·1 | 73·9 | 76·5 | <0·0001 | 82·8 | 81·7 | 0·15 |
| **p-trend‡** | ·· | ·· | ·· | ·· | ·· | ·· | ·· | ·· | <0·0001 | ·· |

Low prevalence, medium prevalence, and high prevalence=residence in low, medium and high gay population prevalence postcodes categories, respectively. *p-trend, time trend 2015-2020; **†**p-value, test for difference between 2020 and 2022; ‡p-trend, test for difference between gay prevalence postcode categories in 2022.

## **Table S2-B:** Proportion of high-risk HIV-negative GBM patients recorded as being on PrEP, overall, and by gay population prevalence postcode category, ACCESS NSW Network 2015-2022

|  | **Year** | | | | | | | | |  |
| --- | --- | --- | --- | --- | --- | --- | --- | --- | --- | --- |
|  | **2015** | **2016** | **2017** | **2018** | **2019** | **p-trend*** | **2020** | **2021** | **2022** | **p-value†** |
| **Overall (n)** | 4434 | 5497 | 6717 | 7561 | 7807 | ·· | 6679 | 5645 | 5796 | ·· |
| On PrEP (n) | 522 | 1957 | 3255 | 4172 | 4039 | ·· | 3648 | 3269 | 3312 | ·· |
| On PrEP (%) | 11·7 | 35·6 | 48·4 | 55·1 | 51·7 | <0·0001 | 54·6 | 57·9 | 57·1 | <0·0001 |
| **Low prevalence (n)** | 1752 | 2238 | 2870 | 3297 | 3595 | ·· | 3215 | 2540 | 2662 | ·· |
| On PrEP (n) | 168 | 707 | 1292 | 1697 | 1789 | ·· | 1716 | 1432 | 1532 | ·· |
| On PrEP (%) | 9·5 | 31·5 | 45·0 | 51·4 | 49·7 | <0·0001 | 53·3 | 56·3 | 57·5 | <0·0001 |
| **Medium prevalence (n)** | 1179 | 1416 | 1756 | 1995 | 2048 | ·· | 1637 | 1514 | 1599 | ·· |
| On PrEP (n) | 146 | 506 | 828 | 1064 | 1009 | ·· | 823 | 846 | 848 | ·· |
| On PrEP (%) | 12·3 | 35·7 | 47·1 | 53·3 | 49·2 | <0·0001 | 50·2 | 55·8 | 53·0 | 0·024 |
| **High prevalence (n)** | 1503 | 1843 | 2091 | 2269 | 2164 | ·· | 1827 | 1591 | 1535 | ·· |
| On PrEP (n) | 208 | 744 | 1135 | 1411 | 1241 | ·· | 1109 | 991 | 932 | ·· |
| On PrEP (%) | 13·8 | 40·3 | 54·2 | 62·1 | 57·3 | <0·0001 | 60·7 | 62·2 | 60·7 | 0·040 |
| **p-trend‡** | ·· | ·· | ·· | ·· | ·· | ·· | ·· | ·· | 0·16 | ·· |

On PrEP=HIV-negative patients recorded as being on PrEP at least once during the calendar year. Low prevalence, medium prevalence, and high prevalence=residence in low, medium and high gay population prevalence postcodes categories, respectively. *p-trend, time trend 2015-2019; **†**p-value, test for difference between 2019 and 2022; ‡p-trend, test for difference between gay prevalence postcode categories in 2022.

# ***Section S3:*** *Community and clinic-collected data on HIV treatment among high-risk HIV positive GBM in New South Wales*

## **Table S3-A:** HIV treatment among HIV positive high-risk GBM (i.e., those who reported condomless anal intercourse with casual partners in the previous six months) overall, and by residence in gay population prevalence postcode category, Sydney Gay Community Periodic Surveys 2008-2022

|  | **Year** | | | | | | | | | | | | | | | |  |
| --- | --- | --- | --- | --- | --- | --- | --- | --- | --- | --- | --- | --- | --- | --- | --- | --- | --- |
|  | **2008** | **2009** | **2010** | **2011** | **2012** | **2013** | **2014** | **2015** | **2016** | **2017** | **2018** | **2019** | **2020** | **p-trend*** | **2021** | **2022** | **p-value**§ |
| **Overall (n)** | 114 | 122 | 144 | 127 | 153 | 115 | 97 | 92 | 115 | 138 | 123 | 100 | 113 | ·· | 74 | 60 | ·· |
| On HIV treatment (n) | 89 | 92 | 113 | 101 | 128 | 95 | 84 | 85 | 102 | 127 | 115 | 94 | 107 | ·· | 71 | 59 | ·· |
| On HIV treatment (%) | 78·1 | 75·4 | 78·5 | 79·5 | 83·7 | 82·6 | 86·6 | 92·4 | 88·7 | 92 | 93·5 | 94·0 | 94·7 | <0·0001 | 95·9 | 98·3 | 0·42 |
| **Low prevalence (n)** | 28 | 24 | 26 | 30 | 38 | 24 | 21 | 25 | 38 | 55 | 49 | 38 | 39 | ·· | 38 | 28 | ·· |
| On HIV treatment (n) | 19 | 20 | 20 | 25 | 33 | 20 | 17 | 25 | 34 | 50 | 44 | 35 | 35 | ·· | 35 | 28 | ·· |
| On HIV treatment (%) | 67·9 | 83·3 | 76·9 | 83·3 | 86·8 | 83·3 | 81·0 | 100 | 89·5 | 90·9 | 89·8 | 92·1 | 89·7 | <0·0001 | 92·1 | 100 | 0·13 |
| **Medium prevalence (n)** | 27 | 35 | 48 | 28 | 35 | 30 | 34 | 19 | 35 | 22 | 26 | 23 | 22 | ·· | 18 | 13 | ·· |
| On HIV treatment (n) | 23 | 25 | 38 | 22 | 26 | 21 | 29 | 15 | 30 | 20 | 25 | 21 | 21 | ·· | 18 | 12 | ·· |
| On HIV treatment (%) | 85·2 | 71·4 | 79·2 | 78·6 | 74·3 | 70·0 | 85·3 | 8·9 | 85·7 | 90·9 | 96·2 | 1·3 | 95·5 | <0·0001 | 100 | 92·3 | 1.0 |
| **High prevalence (n)** | 59 | 63 | 70 | 69 | 80 | 61 | 42 | 48 | 42 | 61 | 48 | 39 | 52 | ·· | 18 | 19 | ·· |
| On HIV treatment (n) | 47 | 47 | 55 | 54 | 86·2 | 54 | 38 | 45 | 38 | 57 | 46 | 38 | 51 | ·· | 18 | 19 | ·· |
| On HIV treatment (%) | 79·7 | 74·6 | 78·6 | 78·3 | 86·2 | 88·5 | 90·5 | 93·8 | 90·5 | 93·4 | 95·8 | 97·4 | 98·1 | <0·0001 | 100 | 100 | 1.0 |
| **p-trend‡** | ·· | ·· | ·· | ·· | ·· | ·· | ·· | ·· | ·· | ·· | ·· | ·· | ·· | ·· | ·· | 0·16 | ·· |

Treat=reported being on combination antiretroviral treatment for HIV. Low prevalence, medium prevalence, and high prevalence=residence in low, medium and high gay population prevalence postcodes categories, respectively. *p-trend, time trend 2008-2020; **†**p-value, test for difference between 2020 and 2022; ‡p-trend, test for difference between gay prevalence postcode categories in 2022. §Fishers exact test

## **Table S3-B*:*** Proportion of high-risk HIV-positive GBM patients recorded on HIV treatment, overall, and by gay population prevalence postcode category, ACCESS NSW Network 2009-2022

|  | **Year** | | | | | | | | | | | | | | |  |
| --- | --- | --- | --- | --- | --- | --- | --- | --- | --- | --- | --- | --- | --- | --- | --- | --- |
|  | **2009** | **2010** | **2011** | **2012** | **2013** | **2014** | **2015** | **2016** | **2017** | **2018** | **2019** | **p-trend*** | **2020** | **2021** | **2022** | **p-value†** |
| **Overall (n)** | 272 | 318 | 339 | 422 | 503 | 595 | 655 | 736 | 791 | 831 | 822 | ·· | 771 | 676 | 525 | ·· |
| On HIV treatment (n) | 193 | 227 | 260 | 340 | 440 | 529 | 609 | 698 | 765 | 820 | 807 | ·· | 765 | 672 | 524 | ·· |
| On HIV treatment (%) | 71·0 | 71·4 | 76·7 | 80·6 | 87·5 | 88·9 | 93·0 | 94·8 | 96·7 | 98·7 | 98·2 | <0·0001 | 99·2 | 99·4 | 99·8 | 0·007 |
| **Low prevalence (n)** | 94 | 115 | 130 | 162 | 206 | 241 | 295 | 339 | 365 | 389 | 358 | ·· | 319 | 270 | 217 | ·· |
| On HIV treatment (n) | 71 | 82 | 102 | 131 | 176 | 215 | 276 | 322 | 357 | 387 | 352 | ·· | 308 | 265 | 216 | ·· |
| On HIV treatment (%) | 75·5 | 71·3 | 78·4 | 80·8 | 85·4 | 89·2 | 93·5 | 94·9 | 97·8 | 99·4 | 98·3 | <0·0001 | 96·5 | 98·1 | 99·5 | 0·20 |
| **Medium prevalence (n)** | 63 | 71 | 74 | 101 | 113 | 142 | 151 | 175 | 179 | 189 | 208 | ·· | 213 | 188 | 150 | ·· |
| On HIV treatment (n) | 43 | 50 | 60 | 89 | 105 | 125 | 136 | 166 | 173 | 185 | 205 | ·· | 212 | 188 | 150 | ·· |
| On HIV treatment (%) | 68·2 | 70·4 | 81·0 | 88·1 | 92·9 | 88·0 | 90·0 | 94·8 | 96·6 | 97·8 | 98·5 | <0·0001 | 99·5 | 100 | 100 | 0·14 |
| **High prevalence (n)** | 115 | 132 | 135 | 159 | 184 | 212 | 209 | 222 | 247 | 253 | 256 | ·· | 227 | 208 | 157 | ·· |
| On HIV treatment (n) | 79 | 95 | 98 | 120 | 159 | 189 | 197 | 210 | 235 | 248 | 250 | ·· | 225 | 207 | 157 | ·· |
| On HIV treatment (%) | 68·7 | 71·9 | 72·5 | 75·4 | 86·4 | 89·1 | 94·2 | 94·5 | 95·1 | 98·0 | 97·6 | <0·0001 | 99·1 | 99·5 | 100 | 0·053 |
| **p-trend‡** | ·· | ·· | ·· | ·· | ·· | ·· | ·· | ·· | ·· | ·· | ·· | ·· | ·· | ·· | 0·29 | ·· |

On HIV treatment=recorded as being on HIV treatment in the previous twelve months. Low prevalence, medium prevalence, and high prevalence=residence in low, medium and high gay population prevalence postcodes categories, respectively. *p-trend, time trend 2009-2019; **†**p-value, test for difference between 2019 and 2022; ‡p-trend, test for difference between gay prevalence postcode categories in 2022.

# ***Section S4:*** *Community and clinic-collected data on undetectable viral load among high-risk HIV positive GBM in New South Wales*

## **Table S4-A*:*** Proportion of high-risk HIV-positive GBM (i.e., those who reported condomless anal intercourse with casual partners in the previous six months) who reported that the result of their last HIV viral load test was undetectable, overall, and by residence in gay population prevalence postcode category, Sydney Gay Community Periodic Surveys 2008-2022

|  | **Year** | | | | | | | | | | | | | | | | |
| --- | --- | --- | --- | --- | --- | --- | --- | --- | --- | --- | --- | --- | --- | --- | --- | --- | --- |
|  | **2008** | **2009** | **2010** | **2011** | **2012** | **2013** | **2014** | **2015** | **2016** | **2017** | **2018** | **2019** | **2020** | **p-trend*** | **2021** | **2022** | **p-value**§ |
| **Overall (n)** | 112 | 121 | 143 | 127 | 154 | 117 | 98 | 93 | 116 | 139 | 123 | 99 | 114 | ·· | 75 | 60 | ·· |
| UVL (n) | 75 | 81 | 112 | 101 | 131 | 97 | 85 | 84 | 101 | 127 | 118 | 93 | 107 | ·· | 69 | 58 | ·· |
| UVL (%) | 67·0 | 66·9 | 78·3 | 79·5 | 85·1 | 82·9 | 86·7 | 90·3 | 87·1 | 91·4 | 95·9 | 93·9 | 93·9 | <0·0001 | 92·0 | 96·7 | 0·72 |
| **Low prevalence (n)** | 29 | 24 | 25 | 30 | 38 | 25 | 22 | 26 | 38 | 56 | 49 | 37 | 39 | ·· | 38 | 28 | ·· |
| UVL (n) | 18 | 17 | 19 | 24 | 32 | 22 | 18 | 23 | 32 | 48 | 46 | 35 | 37 | ·· | 32 | 28 | ·· |
| UVL (%) | 62·1 | 70·8 | 76·0 | 80·0 | 84·2 | 88·0 | 81·8 | 88·5 | 84·2 | 85·7 | 93·9 | 94·6 | 94·9 | <0·0001 | 84·2 | 100 | 0·51 |
| **Medium prevalence (m)** | 27 | 34 | 48 | 28 | 36 | 30 | 34 | 19 | 35 | 22 | 26 | 23 | 22 | ·· | 18 | 13 | ·· |
| UVL (n) | 19 | 24 | 37 | 22 | 28 | 22 | 29 | 19 | 30 | 21 | 25 | 22 | 20 | ·· | 18 | 12 | ·· |
| UVL (%) | 70·4 | 70·6 | 77·1 | 78·6 | 77·8 | 73·3 | 85·3 | 100 | 85·7 | 95·5 | 96·2 | 95·7 | 90·9 | <0·0001 | 100 | 92·3 | 1.0 |
| **High prevalence (n)** | 56 | 63 | 70 | 69 | 80 | 62 | 42 | 48 | 43 | 61 | 48 | 39 | 53 | ·· | 19 | 19 | ·· |
| UVL (n) | 38 | 40 | 56 | 55 | 71 | 53 | 38 | 42 | 39 | 58 | 47 | 36 | 50 | ·· | 19 | 18 | ·· |
| UVL (%) | 67·9 | 63·5 | 80·0 | 79·7 | 88·8 | 85·5 | 90·5 | 87·5 | 90·7 | 95·1 | 97·9 | 92·3 | 94·3 | <0·0001 | 100 | 94·7 | 1.0 |
| **p-trend‡** | ·· | ·· | ·· | ·· | ·· | ·· | ·· | ·· | ·· | ·· | ·· | ·· | ·· | ·· | ·· | 0·38 | ·· |

UVL=men who reported that the result of their last viral load test was undetectable. Low prevalence, medium prevalence, and high prevalence=residence in low, medium and high gay population prevalence postcodes categories, respectively. *p-trend, time trend 2008-2020; **†**p-value, test for difference between 2020 and 2022; ‡p-trend, test for difference between gay prevalence postcode categories in 2022. §Fishers exact test

## **Table S4-B:** Proportion of high-risk HIV-positive GBM patients recorded as having undetectable viral load, overall, and by gay population prevalence postcode category, ACCESS NSW Network 2009-2022

|  | **Year** | | | | | | | | | | | | | | |  |
| --- | --- | --- | --- | --- | --- | --- | --- | --- | --- | --- | --- | --- | --- | --- | --- | --- |
|  | **2009** | **2010** | **2011** | **2012** | **2013** | **2014** | **2015** | **2016** | **2017** | **2018** | **2019** | **p-trend*** | **2020** | **2021** | **2022** | **p-value†** |
| **Overall (n)** | 269 | 318 | 337 | 415 | 487 | 586 | 649 | 724 | 779 | 808 | 795 | ·· | 755 | 660 | 522 | ·· |
| UVL (n) | 184 | 225 | 247 | 316 | 399 | 510 | 601 | 692 | 756 | 780 | 759 | ·· | 736 | 649 | 518 | ·· |
| UVL (%) | 68·4 | 70·8 | 73·3 | 76·1 | 81·9 | 87·0 | 92·6 | 95·6 | 97·0 | 96·5 | 95·5 | <0·0001 | 97·4 | 98·3 | 99·2 | <0·0001 |
| **Low prevalence (n)** | 92 | 115 | 128 | 158 | 202 | 237 | 291 | 334 | 360 | 376 | 338 | ·· | 319 | 270 | 217 | ·· |
| UVL (n) | 61 | 77 | 97 | 122 | 158 | 205 | 266 | 319 | 349 | 362 | 321 | ·· | 308 | 265 | 216 | ·· |
| UVL (%) | 66·3 | 66·9 | 75·7 | 77·2 | 78·2 | 86·5 | 91·4 | 95·5 | 96·9 | 96·2 | 94·9 | <0·0001 | 96·5 | 98·1 | 99·5 | 0·003 |
| **Medium prevalence (n)** | 63 | 71 | 74 | 100 | 109 | 141 | 150 | 172 | 177 | 185 | 205 | ·· | 212 | 184 | 149 | ·· |
| UVL (n) | 40 | 54 | 59 | 84 | 95 | 122 | 139 | 165 | 173 | 176 | 193 | ·· | 207 | 183 | 147 | ·· |
| UVL (%) | 63·4 | 76·0 | 79·7 | 84·0 | 87·1 | 86·5 | 92·6 | 95·9 | 97·7 | 95·1 | 94·1 | <0·0001 | 97·6 | 99·4 | 98·6 | 0·032 |
| **High prevalence (n)** | 114 | 132 | 135 | 157 | 176 | 208 | 208 | 218 | 242 | 247 | 252 | ·· | 224 | 206 | 156 | ·· |
| UVL (n) | 83 | 94 | 91 | 110 | 146 | 183 | 196 | 208 | 234 | 242 | 245 | ·· | 221 | 201 | 155 | ·· |
| UVL (%) | 72·8 | 71·2 | 67·4 | 70·0 | 82·9 | 87·9 | 94·2 | 95·4 | 96·6 | 97·9 | 97·2 | <0·0001 | 98·6 | 97·5 | 99·3 | 0·13 |
| **p-trend‡** | ·· | ·· | ·· | ·· | ·· | ·· | ·· | ·· | ·· | ·· | ·· | ·· | ·· | ·· | 0·78 | ·· |

UVL=patients recorded as having undetectable viral load (<200 RNA copies/mm^3^) at their most recent test in the previous 12 months. Low prevalence, medium prevalence, and high prevalence=residence in low, medium and high gay population prevalence postcodes categories, respectively. *p-trend, time trend 2009-2019; **†**p-value, test for difference between 2019 and 2022; ‡p-trend, test for difference between gay prevalence postcode categories in 2022.
